# Socioeconomic inequalities in post-natal health checks for the newborn in Vietnam

**DOI:** 10.1186/s12939-019-1029-8

**Published:** 2019-08-16

**Authors:** Nguyen Duc Lam, Nguyen Duy Anh, Nguyen Thi Thu Ha, Truong Quang Vinh, Vu Thi Mai Anh, Vu Duy Kien

**Affiliations:** 1 Hanoi Medical University, No1. Ton That Tung Street, Hanoi, Vietnam; 2 Hanoi Obstetrics and Gynecology Hospital, No. 929 La Thanh Street, Ba Dinh District, Hanoi, Vietnam; 3 Vietnam National University, No 144 Xuan Thuy Street, Cau Giay District, Hanoi, Vietnam; 4 Health Strategy and Policy Institute, A36 Lane, Ho Tung Mau St, Cau Giay District, Hanoi, Vietnam; 5 OnCare Medical Technology Company Limited, No. 77/508 Lang Street, Hanoi, Vietnam

**Keywords:** Post-natal, Health checks, Newborn, Inequalities, Concentration index, Vietnam

## Abstract

**Background:**

The newborn and child death associated with inadequate post-natal health checks continued to be a significant issue across the world. This study aimed to assess the socioeconomic inequalities in post-natal health checks for the newborn in Vietnam in 2014.

**Methods:**

We used the secondary data from the Multiple Indicator Cluster Survey in 2014. We included women aged 15–49 years who had a live birth within two years of the time of the interview. We estimated the concentration index to measure socioeconomic inequalities post-natal health checks for the newborn. We conducted multiple logistic regression analysis to identify factors associated with post-natal health checks for the newborn.

**Results:**

Overall, the proportion of post-natal health checks for the newborn in Vietnam was 89.1%. The concentration index of post-natal health checks for the newborn was positive at 0.06. It indicated that the newborns in the rich households were more likely to get post-natal health checks as compared to in the poor households. The common factors significantly associated with the higher percentage of post-natal health checks for the newborn were women belonging to the Kinh and Hoa ethnic, higher education, and wealthier groups.

**Conclusion:**

Socioeconomic inequalities in post-natal health checks for the newborn in Vietnam were not strong, but it still existed. Thus, we recommended that policy efforts to increase access to post-natal health services for poor women. In addition, there is a need to improve access to post-natal health services for women belonging to minor ethnic group and low education.

## Background

The World Health Organization (WHO) developed a guideline on post-natal care for the mother and newborn, which considered the post-natal period as the first 6 weeks after birth [1]. The post-natal period is a vital stage that impacts on lives of mothers and newborn babies. Evidence shows that most deaths of mothers and newborns occur during this period [1]. It was estimated that more than a million newborn deaths occurred on the first day of their life every year [2–4]. Most of the newborn deaths were observed in low- and middle- income countries, particular in South Asia and Sub-Saharan Africa [4]. The deaths of newborns associated with inadequate post-natal health checks continued to be a significant issue across the world, particular in low- and middle-income countries [5].

There has been a grown concern related to inequalities in post-natal healthcare checks in various regions of the world [6]. The post-natal health checks were shown to favor the rich in Sri Lanka. In addition, the residence and the education level of women contributed significantly to the socioeconomic inequality in post-natal health checks [7]. A meta-analysis study revealed significant differences within the low- and middle-income countries based on socioeconomic status and urban and rural setups [6]. Among these, few worth mentioning is the lack of nearby healthcare facility for post-natal health care, literacy rate, number of pregnancies, and religion affects post-natal care check [8]. Studies from several regions showed that healthcare facilities were relatively distant, and women did not have appropriate access [8, 9].

In Vietnam, post-natal care services are provided by the primary healthcare system, which includes district hospitals and commune health stations. With the effort of the Vietnam healthcare system, the infant mortality rate per 1000 livebirths decreased to 14.5‰ in 2016 as compared to 36.7‰ in 2000 [10, 11]. The Vietnamese Health Ministry is cognizant of the issues related to post-natal health checks and their overall impact [12, 13]. However, no data on post-natal care was collected systematically before the year 2014 [14]. The information of post-natal care is important to monitor progress on maternal, newborn, and child health interventions [15]. Moreover, the was no study on post-natal health checks that took into account the issue of socioeconomic inequality. Thus, this study is aimed at assessing the socioeconomic inequalities in post-natal health checks for the newborn. In addition, the purpose of this study is to explore the associated factors with post-natal health checks for the newborn in Vietnam.

## Methods

### Data sources

Data were extracted from the fifth Vietnam Multiple Indicator Cluster Survey (MICS) conducted in 2014 [14]. The MICS is an initiative of the United Nations Children’s Fund (UNICEF) to assists countries to monitor the situation of children and women. In Vietnam, this was a nationally representative survey, which was conducted every 4 or 6 years. Five rounds of MICS were done from 1996, and the latest round of MICS was in 2014. The survey used a two-stage sampling approach, which was done for region, urban and rural areas. All geographic regions of Vietnam were selected, and urban and rural areas were identified as the main sampling strata in each region. In MICS 2014, six geographic regions were determined, and it was estimated that the sample needed in each region was 1700 households. The number of households selected in each cluster was 20 households, and it was considered as enumeration area (EA). In each region, it was estimated that the survey needed to select 85 sample clusters. To identity EA in each region, the probability proportional to size method was applied. Within each EA, a random systematic selection method was used to select 20 households. The total sample size for MICS 2014 was estimated as 10,200 households. In the survey, three sets of questionnaires were used, including the questionnaires to collect information on household status, women aged between 15 and 49 years old, and children under 5 years old. Detail information about the MICS 2014 was provided elsewhere [14]. In the MICS 2014, it was the first time that the information about post-natal health checks for the newborn was included. In this study, we focused only on the women who had a live birth in the last 2 years at the time of being interviewed, and the women with complete information. Thus, the total number of women included in this study was 1473 women.

### Variables

Post-natal health checks for the newborn were defined as any health check performed in a health facility, at home following a birth or care visits within 2 days of delivery, and included preventive care services. The main outcome variable in this study was the binary variable of whether or not a newborn was provided with a health check, as defined above. Independent variables relating to the mother included age group (less than 20, 20–34 or 35–49 years), place of residence (urban or rural), ethnicity (minorities Kinh or Hoa), education level (primary or lower, lower secondary, and upper secondary and tertiary) and type of delivery (Caesarian-section or vaginal birth).

### Measurement of socioeconomic status

The wealth asset index was used as a proxy for socioeconomic status. The wealth assets index was calculated using principal component analysis (PCA). To construct the wealth assets index, the PCA was performed by using the information of household goods, household characteristics, water, and sanitation. In detail, the household goods were considered if a household had electricity, radio, television, telephone, refrigerator, bed, table, sofa, fan, computer, air-conditioner, gas cooker, electric cooker, electric cooker, washing machine, tractor, car or ship and boat with motor. The household characteristics included main materials to build the floor, the roof, and the exterior walls. The information about water and sanitation used was about the water source and sanitation facility that the household used daily. In addition, other indicators might be used to construct the wealth asset index, which included the size of the land, the ownership of the land or number of animals. The PCA was done to generate the factor score for urban and rural areas, and then the combined factor was estimated based on the weight for the total sample. Each household in the total sample was assigned a wealth asset score based on the assets that the household had. The details of the method used for estimating the wealth asset index are described elsewhere [14]. Based on the wealth asset score, a household in the sample was ranked from the poorest to the richest. Furthermore, the survey households were divided into five equal quintiles, ranging from poorest to richest groups.

### Measurement of socioeconomic inequalities

The magnitude of socioeconomic inequality in post-natal health checks for the newborn was assessed using a concentration index [16]. The equation for the concentration index was as follows:

$$ \mathrm{C}=\frac{2}{\upmu}\mathrm{Cov}\left(\mathrm{h},\mathrm{r}\right) $$

Where C is the estimated concentration index, is the proportion of post-natal health checks for the newborn in the study population, h is the status of the post-natal health check for the newborn (whether or not a post-natal health check was provided), and r is the fractional rank of the socioeconomic status of the mother’s household. The concentration index ranges from − 1 to + 1. If the concentration index is zero, there would be no socioeconomic-related inequality in post-natal health checks for the newborn. If the concentration index is negative, the proportion of post-natal health checks will take higher values among poorer households. On the other hand, if the concentration index is positive, the proportion of post-natal health checks will take higher values among wealthier households.

### Statistical analysis

Descriptive statistics were used to estimate the proportion of newborn receiving post-natal health checks. The proportion and 95% confidence interval (CI) for post-natal health checks for the newborn were calculated for each independent variable related to the mother, including age group, place of residence, education, type of delivery and socioeconomic status. Multivariable logistic regression analysis was conducted to identify factors associated with post-natal health checks for the newborn. All statistical analyses in this study were conducted by STATA® 13.1, using the weighting of variables for women in the dataset. We set the level of statistical significance at p less than 0.05.

## Results

### Post-natal health checks

Table 1 shows the proportion of post-natal health checks for the newborn in Vietnam in 2014 for each independent variable analyzed. Overall, the proportion of post-natal health checks for the newborn in Vietnam was 89.1% (95% CI: 87.3–90.7). The proportion of post-natal health checks in urban areas (94.3, 95% CI: 91.7–96.1) was higher than that in rural areas (87.0, 95% CI: 84.6–89.0). The proportion of post-natal health checks was higher among the Kinh/Hoa ethnicity than among minority ethnic groups. Post-natal health care checks were also more frequent among women with higher education, higher socioeconomic status, and those who received a Caesarian (C)-section.

**Table 1:** Frequency of post-natal health checks for the newborn in Vietnam in 2014 by selected independent variables

	Post-natal health checks for the newborn (weighted) % (95% CI)
Age group (year)	
Less than 20	84.9 (81.1–87.9)
20–34	91.3 (89.1–93.1)
35–49	89.5 (83.6–93.5)
Area	
Rural	87.0 (84.6–89.0)
Urban	94.3 (91.7–96.1)
Ethnicity	
Minorities	61.0 (55.1–63.6)
Kinh/Hoa	94.8 (93.2–96.1)
Education	
Primary or less	68.3 (62.2–73.8)
Lower secondary	89.7 (86.5–92.3)
Upper secondary and tertiary	96.0 (94.1–97.4)
Type of delivery	
Vaginal birth	86.1 (83.8–88.1)
C-section	96.9 (94.4–98.4)
Socioeconomic status (quintile)	
Poorest	61.3 (55.0–67.3)
Near poor	88.7 (83.9–92.2)
Middle	92.8 (88.6–95.5)
Richer	95.8 (92.6–97.7)
Richest	96.4 (93.1–98.2)
Total	89.1 (87.3–90.7)

### Socioeconomic inequalities in post-natal health checks

**Fig. 1:**
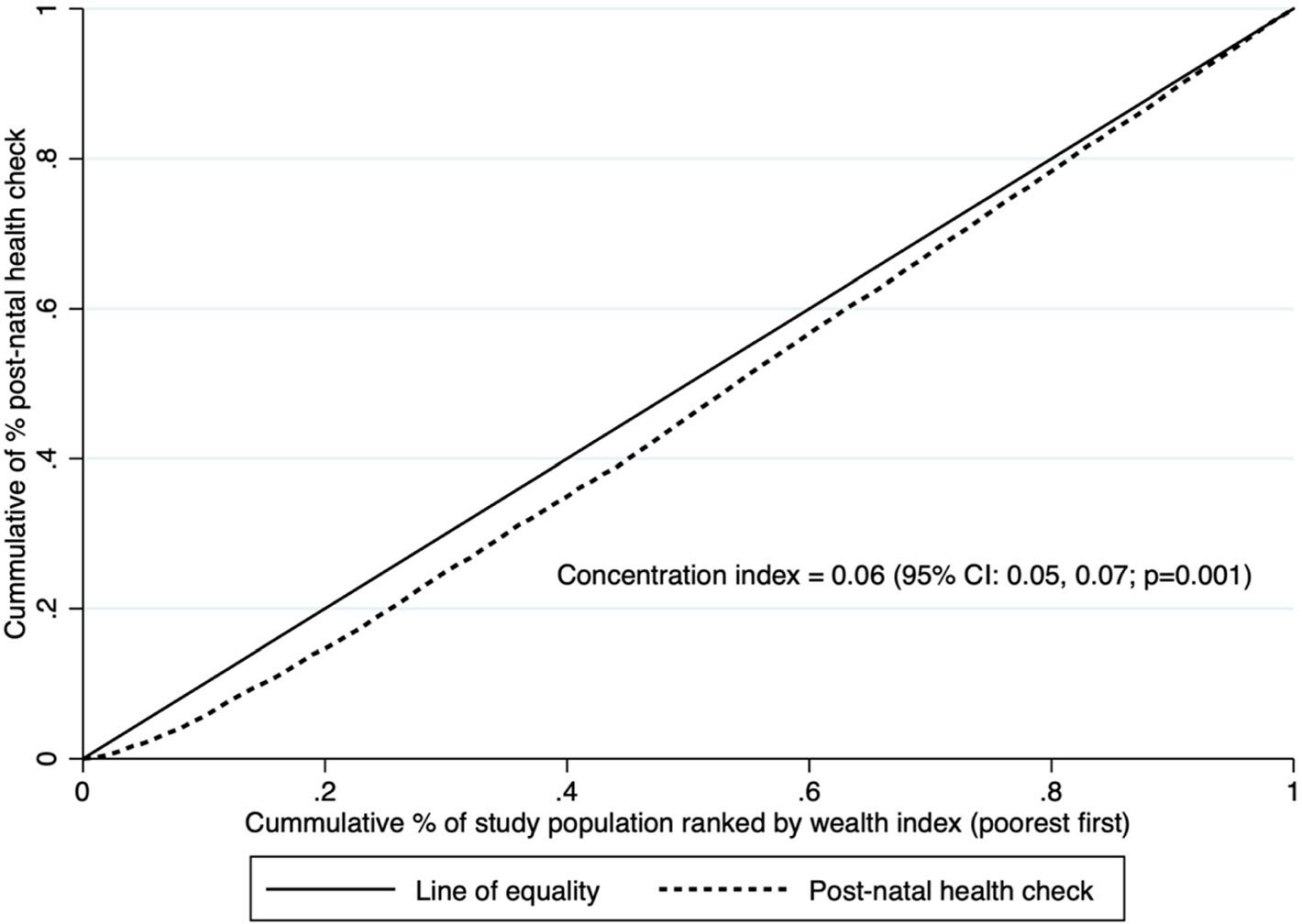
Concentration curve and concentration index for wealth-based inequality in post-natal health checks for the newborn in Vietnam in 2014

Figure 1 illustrates the concentration curve of post-natal health checks for the newborn in Vietnam in 2014. The concentration curve was under the line of equality, indicating that post-natal health checks were provided more frequently to the newborn in wealthier households. The concentration index was positive at 0.06 (95% CI: 0.05–0.07, *p* = 0.001), further confirming this result.

### Associated factors for post-natal health checks

Table 2 presents the results of multivariable logistic regression analysis to assess the association between post-natal health checks for the newborn and selected independent variables. The post-natal health checks significantly associated with women in wealthier households. The proportion of post-natal health checks increased among households of near-poor (OR = 2.2, 95% CI: 1.3–3.7), middle (OR = 2.4, 95% CI: 1.3–4.6), richer (OR = 3.5, 95% CI: 1.6–7.6) and richest (OR = 2.9, 95% CI: 1.2–7.3) as compared with the poorest households. The newborn of women with lower secondary school education (OR = 2.1, 95% CI: 1.3–3.2), upper secondary and tertiary school education (OR = 3.7, 95% CI: 2.1–6.4) had the significantly higher proportion of post-natal health checks as compared with the newborn of women with primary school education or less. The proportion of post-natal health checks for the newborn was higher among Kinh/Hoa ethnic groups (OR = 4.3, 95% CI: 2.7–6.9) than among ethnic minority groups. The newborn of women receiving a C-section had a higher proportion of post-natal health checks (OR = 2.7, 95% CI: 1.3–5.4) as compared with that of women who underwent vaginal birth.

**Table 2:** Associated factors for post-natal health checks for the newborn of women aged 15–49 in Vietnam: multivariable logistic regression analysis

	Post-natal health checks for newborn	
	OR (95% CI)	p
Age group (year)		
Less than 20	Ref.	
20–34	1.0 (0.6–1.6)	0.96
35–49	0.9 (0.5–2.1)	0.98
Area		
Rural	Ref.	
Urban	1.4 (0.8–2.4)	0.24
Ethnicity		
Minorities	Ref.	
Kinh/Hoa	4.3 (2.7–6.9)	0.001
Education		
Primary or less	Ref.	
Lower secondary	2.1 (1.3–3.2)	0.001
Upper secondary and tertiary	3.7 (2.1–6.4)	0.001
Type of delivery		
Vaginal birth	Ref.	
C-section	2.7 (1.3–5.4)	0.01
Socioeconomic status (quintile)		
Poorest	Ref.	
Near poor	2.2 (1.3–3.7)	0.01
Middle	2.4 (1.3–4.6)	0.01
Richer	3.5 (1.6–7.6)	0.01
Richest	2.9 (1.2–7.3)	0.02

Note. *OR* odds ratio, *CI* confidence interval

## Discussion

This study was the first of its kind to collect information about socioeconomic inequalities related to post-natal health checks for the newborn in Vietnam. The concentration index as a measure of socioeconomic inequalities indicated that post-natal checks were of higher proportion in the wealthy households, compared with the poor ones. The results demonstrated that besides socioeconomic status, type of delivery, education, and ethnicity significantly associated with the proportion of post-natal health checks of newborns. These findings provide new data on newborns’ post-natal checks in Vietnam and contribute to the understanding of socioeconomic differentials in their utilization.

As literature data indicate, the highest risk of deaths for newborns is either at childbirth or in the period after birth. What is striking is the fact that the vast majority of these deaths occurs in the first 24 h following delivery [4]. Receiving adequate post-natal care is, thus, crucial for improving the health status of both mothers and newborns [17–19]. Although the World Health Organization recommends routine post-natal care provided to all mothers and newborns, in practice, this seems not to be the case [20].

The proportion of post-natal checks of newborns in our study was 89.1%. In comparison with the studies in other developing parts of the world, we obtained a higher level of post-natal health checks. A study conducted in Indian population indicated that only 45% of newborns received health checks within the first 24 h from delivery, while 62% of babies had checks eventually within the early 10 days of life [21]. Another study in Sierra Leone reported that 77.4% of newborns born in a facility received health checks within 2 days of delivery, while this proportion was 59.5% for babies born at home [22]. Data from Zambia health and demographic survey reported that on average, the was 55% of newborns received post-natal checks. If it was disaggregated by delivery place, 58% of newborns born at healthcare facilities had health checks, and 48% of newborns delivered at home [20]. Recently, a study in Sri Lanka reported that the proportion of mothers who received post-natal health checks were 82.6% at the national level [7]. In comparison with these studies, Vietnam healthcare system seems to provide high coverage of post-natal health checks. Still, our data indicated some significant differences between the different subpopulation groups.

Significant socioeconomic inequalities in access to post-natal checks, observed in our study, have been reported in previous studies as well. The mentioned study in Indian population showed an inadequate level of post-natal checks, especially in women and newborns of lower socioeconomic status [21]. Similarly, some also detected positive concentration index of post-natal checks [1, 7]. In our study, the proportion of health checks in rural areas was 87% while it amounted over 94% in urban parts. Accordingly, Zambian health and the demographic survey found that newborns delivered in rural areas were less likely to receive post-natal checks than those born in urban areas [20].

Large demographic and health study investigated equality in the use of maternal and newborn health services in five African countries (Burundi, Kenya, Rwanda, Tanzania, and Uganda). The authors used data from nearly 10 thousand health facilities to assess post-natal health checks within 48 h from delivery [23]. They found that increasing wealth and education correlated with the highest odds of having maternal and newborns’ health services care. More precisely, those in the wealthiest quintile had 1.5 to 2 times more chance of obtaining post-natal health checks [23]. Like in our study, those living in rural areas had lower chances of receiving health checks (10% lower in comparison with urban areas). Further, those with the highest education had two to three times increased chances of having post-natal checks compared to those with no education. Similarly, the survey identified education as one of the factors significantly associated with the odds of receiving post-natal checks for newborns. The newborn of women with secondary (lower or upper) and tertiary school education received a significantly higher proportion of post-natal health checks comparing with the newborn of women with only primary school education or less [23].

Another factor that associated with the proportion of post-natal checks in our study was the type of delivery. We observed a higher percentage for newborns delivered by Caesarean, i.e., C-section in comparison of those born by vaginal birth (odds ratio of 2.7). To the best of our knowledge, not many studies reported the impact of the delivery type on the proportion of post-natal checks of newborns. In our study, we would like to explore the effect of the delivery type because the rate of C-section deliveries was more likely to increase worldwide [24]. Literature data on C-section relation with post-natal health services are not only limited for the newborns, but the mothers as well. Similarly to our results, one survey conducted in rural Tanzania reported that women that had a complicated mode of delivery (such as C-section) were more likely to receive post-natal care from health facilities [25].

In this survey, we identified ethnicity as a significant factor associating with the proportion of the post-natal health check for the newborn, with a lower portion observed among minority ethnic groups. The difference could be that the minority ethnic groups had different attitudes and health-seeking behaviors related to post-natal healthcare check for the newborn. In addition, the minority ethnic women often lived in remote and mountainous areas, so they were less likely to get access to the post-natal healthcare check for the newborn. Ethnic disparities in the utilization of maternal health care have been investigated elsewhere as well. Ghana Maternal Health Survey explored the impact of ethnicity on various aspects of the maternal health care system, including post-natal health checks. The authors included the women having delivery in 5 years preceding the survey and reported the lower proportion of health care obtained in women of minor ethnicity, caused by the higher percentage of home deliveries in these women [26]. Another survey explored disparities in the utilization of health care services between Hispanic and Non-Hispanic White Women in Rhode Island. As this survey reported, children of Hispanic women had higher odds of not having a post-natal (within 1 week) checks or any baby care. The authors named lack of health insurance, education, language, and other social barriers as some of the contributing factors of decreased healthcare access and utilization in the Hispanic population [27].

Despite the importance and novelty of our findings, we are well aware of the limitations of this study. Probably, the primary limitation of our study was the recall bias because some women might not remember the events after they delivered. In this study, we did not include the place of birth into analysis because only 8.7% of women reported that they gave birth at home. Moreover, among those who delivered at home, only 5.5% of women got the post-natal health check for the newborn, so in total, only 0.5% of women got the service when the delivered at home. A further study with a bigger sample size should be conducted to understand more about the impact of the place of birth on the post-natal health check for the newborn. We expected that giving birth at home would be less likely to get the post-natal health check for the newborn service. In Vietnam, health insurance coverage was more than 80% in 2016 [11]. However, health insurance only covers the package of giving birth at a healthcare facility. Thus, if a woman gives birth at a healthcare facility, she may get post-natal check for her child. Because the dataset of MICS 2014 did not collect the information about health insurance, so we could not analyze the impact of health insurance in this study. In addition, a further study should be done to decompose the concentration index, which helps to identify the dominant factors that contribute to the socioeconomic inequality in the post-natal health check for the newborn. Finally, this was a cross-sectional study, thus, it did not allow us to conclude on the causal relationship.

## Conclusion

Overall, we observed socioeconomic inequalities in post-natal health checks for the newborn existed, but it was not strong in Vietnam. Despite the high portion of post-natal health checks, here presented socioeconomic inequalities in receiving post-natal health care should be carefully addressed. Policy efforts should be more targeted towards vulnerable groups, including rural parts, poor households, women with low education and minor ethnic groups, to reduce socioeconomic inequalities in post-natal health checks for the newborn.

## Data Availability

Not applicable.
